# Open Globe Injuries: Classifications and Prognostic Factors for Functional Outcome

**DOI:** 10.3390/diagnostics11101851

**Published:** 2021-10-08

**Authors:** Christian Steffen Mayer, Lukas Reznicek, Isabella Diana Baur, Ramin Khoramnia

**Affiliations:** 1 Department of Ophthalmology, Heidelberg University, 69120 Heidelberg, Germany; Christian.Mayer@med.uni-heidelberg.de (C.S.M.); Isabella.Baur@med.uni-heidelberg.de (I.D.B.); 2 Department of Ophthalmology, Ludwig-Maximilians-University, 80336 Munich, Germany; Lukas-Reznicek@gmx.de

**Keywords:** open globe injuries, ocular trauma, prognostic factor

## Abstract

This paper explored epidemiology and evaluation of posterior segment involvement as prognostic factors for functional outcome of patients with open globe injuries. A retrospective analysis of 151 patients with open globe injuries was conducted. Pre- and postoperative-corrected distance visual acuity (CDVA), epidemiologic data, classification of the injuries including the ocular trauma score (OTS), performed surgeries, intraocular pressure (IOP) and correlation analyses between OTS and postoperative CDVA were obtained. A total of 147 eyes were included in the study. Mean age was 42.9 ± 22.2 years, 78.2% were male, and 36.7% of injuries occurred in the workplace. Thirty-eight patients (25.9%) had intraocular foreign bodies. Concerning injury location, 51.7% of the injuries were located in zone I (cornea, corneoscleral limbus), 15.0% in zone II (up to 5 mm posterior the sclerocorneal limbus) and 32.0% in zone III (posterior of zone 2). Affected structures were eyelids (17.7%), cornea (74.8%), iris (63.9%), lens (56.5%), sclera (48.3%), retina (47.6%) and optic nerve (19.7%). Mean preoperative CDVA was 1.304 ± 0.794 logMAR and 1.289 ± 0.729 logMAR postoperatively (*p* = 0.780). Patients with posterior segment involvement had significantly worse postoperative CDVA than patients without (1.523 ± 0.654 logMAR vs. 0.944 ± 0.708 logMAR, *p* < 0.01). Predictive factors for good visual outcome of open globe injuries are good initial CDVA and ocular trauma affecting only zone I and II.

## 1. Introduction

Epidemiologic data of open globe injuries are rare in Europe. In Germany, the prevalence remains low, with 3 injuries per 100,000 inhabitants a year. The frequency of those severe eye injuries has remained basically constant in the past [1].

Generally speaking, the classification of open globe injuries depends on the injury mechanism and damage of relevant structures of the eye [2,3,4]. In addition, depending on the extent of the primary trauma, various complications such as retinal detachment, secondary glaucoma or secondary traumatic cataract can occur in the years following the trauma. Due to this fact, many of those patients need follow up check-ups for many years after the initial operative treatment.

In the last two decades however, advances in the surgical care of open globe injuries have improved the outcome significantly [5,6,7]. Nevertheless, the prognosis of those severe eye injuries, especially involving the posterior segment, remains poor and, in most cases, unpredictable [8,9,10]. Kuhn et al. were the first to categorize open globe injuries into different categories according to an ocular trauma score (OTS, Table 1) in order to be able to look for possible prognostic factors [4,11]. The OTS is a number between 0 and 100 and is supposed to have a predictive value for the functional outcome (corrected distance visual acuity, CDVA) of the injured eye; it is calculated from the initial CDVA after the trauma and before surgery and also includes additional variables such as “globe rupture” or “relative afferent pupillary defect”. Details can be seen in Table 1.

The goal of this study was to collect enough epidemiologic data of ocular traumas to evaluate the involvement of the posterior segment as a potential negative prognostic factor for the functional outcome of those patients.

## 2. Materials and Methods

We collected retrospective data from 151 patients who suffered from open globe injuries and were referred to the department of Ophthalmology at the Technical University of Munich in Germany across 7 years.

Open globe injuries are defined as a full-thickness wound of the eyewall with sharp or pointed objects (penetrating and/or perforating injuries), intraocular foreign bodies as well as globe ruptures. Therefore, blunt ocular traumas were distinguished from open globe injuries and were excluded.

In each case, patient data as well as circumstances of the accidents were recorded and documented. If feasible, a full ophthalmic examination was performed including the patient’s history with the mechanism of injury, the location of the wound, and a description of damaged eye structures with the presence or absence of an afferent pupillary defect (APD), cataract, iris prolapse, vitreous prolapse, retinal detachment (RD) or intraocular foreign bodies (IOFB) [12]. In addition, corrected distance visual acuity (CDVA) and intraocular pressure (IOP) were obtained. X-rays to exclude orbital fractures or foreign bodies were additionally obtained from each patient.

### 2.1. Classification of Open Globe Injuries

Open globe injuries were classified into A: “type of injury”; B: the grade (visual acuity prior to surgical care); C: the location of the wound (“zone classification I-III”) [4,13]; and D: OTS Category

The calculation of the OTS and classification of the injuries were performed by an experienced investigator (CM).

**A:** The type of injury was divided into subgroups depending on the mechanism: globe ruptures (caused by blunt trauma), globe penetration and perforation (caused by a sharp object) and penetrating injuries with intraocular foreign bodies (IOFB)

**B:** The grade of an ocular trauma is part of the ocular trauma score (OTS), originally published to estimate the prognosis for the visual outcome in ocular trauma of all kinds [11] (see Table 1). Grades 1–5 correspond to the initial visual acuity prior to surgery as indicated in the very left column of the OTS calculation table. Grade 6 refers to cases in which the initial visual acuity remained unknown.

**Figure 1:**
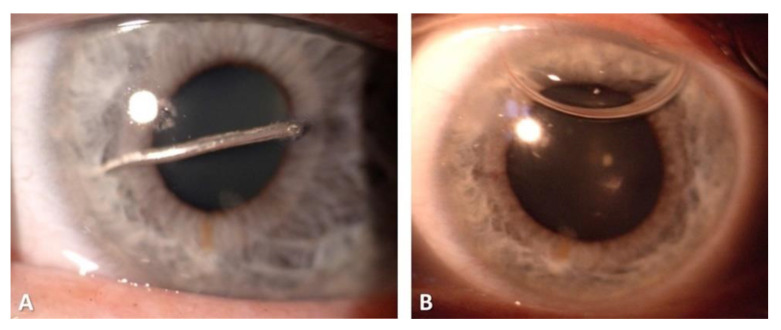
Zone I injury with corneal penetration by a nail. Before (**A**) and after surgery (**B**).

**Figure 2:**
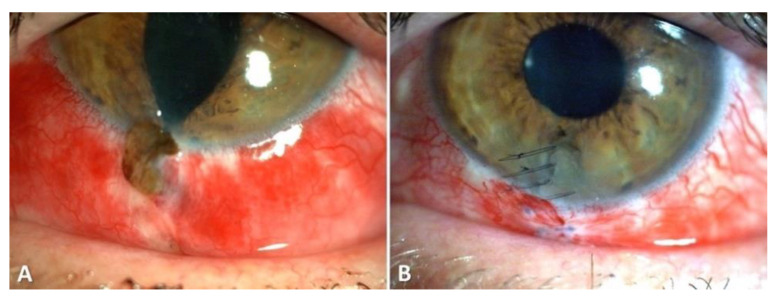
Example of a zone II open globe injury: Corneal dehiscence caused by a broken goggle with a consecutive iris prolapse. Setting before surgery (**A**) and after iris reposition and wound closure (**B**).

**Figure 3:**
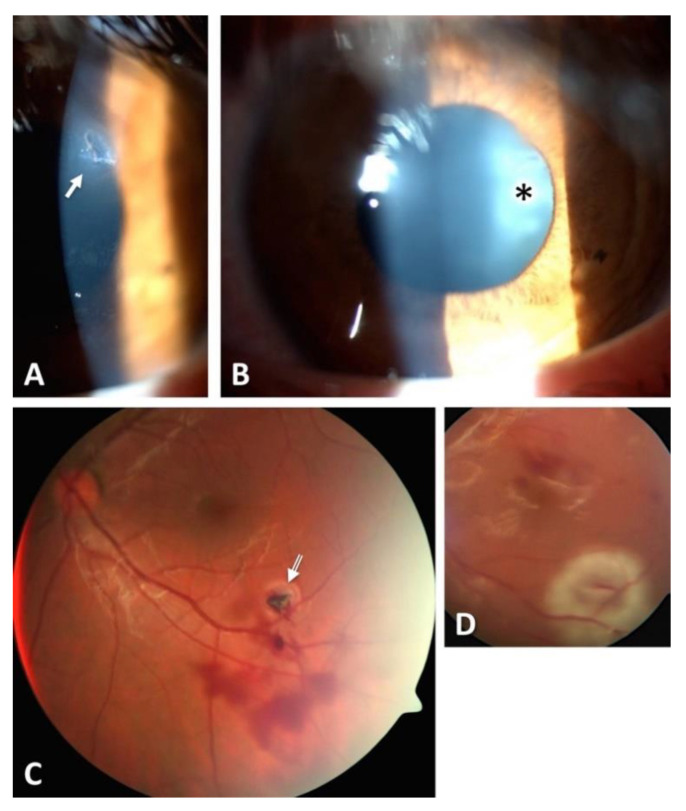
One case with a zone I injury. Penetration of a foreign body at the limbus. Open globe injury with small corneal entrance wound (arrow) (**A**), punctured lens (asterisk) (**B**) and retinal impact (**C**) by a small metallic foreign body (arrow). Intraoperative situation after removal of the foreign body, laser retinopexy and silicone-oil filling (**D**).

**Figure 4:**
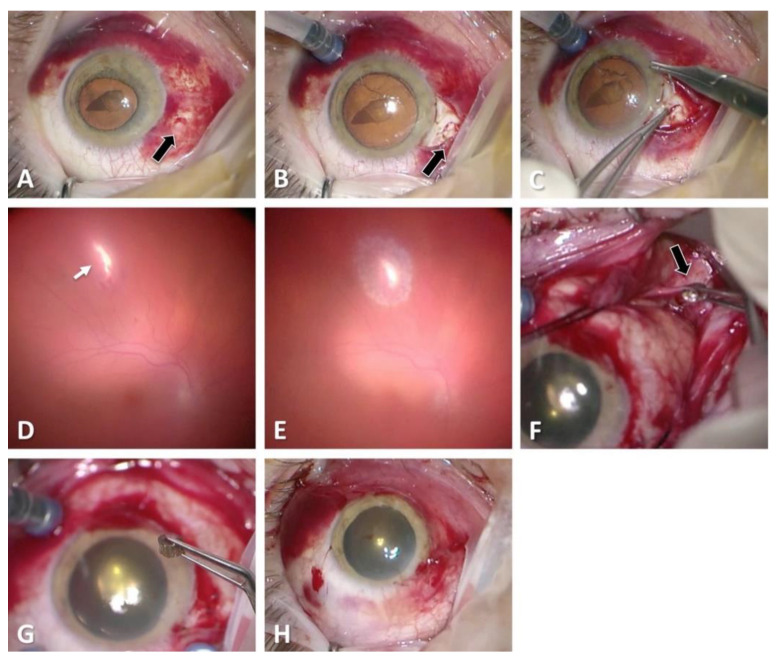
Zone III perforating open globe injury: Preoperative findings of the anterior segment with a self-closed entry of the foreign body 2 mm posterior the corneoscleral border (black arrow) (**A**–**C**). Vitrectomy and intraoperative situation (**D**,**E**): After removing the vitreous hemorrhage, a secondary posterior laceration is revealed (white arrow). The foreign body is not detectable from inside. Additional opening of the conjunctiva and inspection of the posterior sclera (**F**). Removal of the foreign body located at the posterior pole (black arrow) (**F**). End of surgery (**G**–**H**).

**Figure 5:**
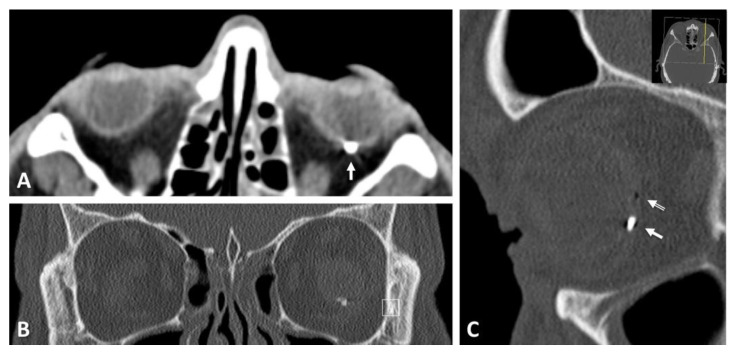
Computed tomographic images from the same patient mentioned in Figure 4. Frontal (**A**), axial (**B**) and sagittal (**C**) images, location of the metallic foreign body at the posterior pole (white arrow).

**C:** Regarding zone classification, the subgroups are categorized according to the location of the most posterior point of the full-thickness wound of the globe: in group I, the most posterior point is isolated to the cornea (including the corneoscleral limbus), as shown in Figure 1; in group II, up to 5 mm posterior of the corneoscleral limbus into the sclera, as shown in Figure 2 and Figure 3; and in group III, posterior of that in group II, as shown in Figure 4 and Figure 5.

**D:** The OTS was calculated according to the published guidelines [11]. In our study, all eyes suffered from an open globe injury; therefore, OTS category 5 was never reached. If, for example, a patient had a vision of only hand movement (equals 70 points) with a diagnosed globe rupture (minus 23 points) and retinal detachment (minus 11 points), the total sum of points is 36 points, thus reaching category 2 according to the OTS score (Table 1).

Primary surgical intervention including ocular reconstruction was planned for the soonest time possible. All interventions were conducted under total intravenous anesthesia. All patients received immediate systemic antibiotics for endophthalmitis prophylaxis (Cefuroxime 1.5 g three times/day for one week) and received a tetanus shot if necessary [6,14,15,16].

### 2.2. Statistical Analysis

Data were collected and analyzed using SPSS 22.0 (SPSS Inc., Chicago, IL, USA) and are presented as arithmetic mean values ± standard deviations. Parametric (*t*-test) and non-parametric (Wilcoxon test) analyses were performed. A *p*-value ≤ 0.05 was considered statistically significant.

## 3. Results

In total, we were able to include 147 eyes with open globe injuries referred to the clinic. Patients’ characteristics, age categories and percentage of work-related accidents can be seen in Table 2.

The various classifications of all globe injuries, including the calculation of the OTS, can be seen in Table 3. The most frequent mechanism of injury was a penetrating trauma (*n* = 58, 39.5%). Applying the zonal classification, 76 (51.7%) of the open globe injuries were zone I injuries, 22 (15.0%) zone II injuries and 47 (32.0%) zone III injuries, as can be seen in Table 3 and Figure 1, Figure 2, Figure 3 and Figure 4. The most common damaged eye structures were cornea (*n* = 110, 74.8%) followed by iris (*n* = 94, 63.9%), vitreous (*n* = 88, 59.9%) and lens (*n* = 83, 56.5%) (Table 4).

Thirty-eight of 147 eyes (25.9%) had intraocular foreign bodies (IOFBs), as shown in Figure 3. Object materials were mainly metal (84.2%) followed by glass (*n* = 4, 10.5%) and organic material (*n* = 2, 5.3%) (Table 4). In 36 cases (94.7%), the IOFB could be removed successfully, whereas in 2 cases (5.3%), the foreign body had to be left in the globe. All patients received primary care surgery; the revised ophthalmic structures during primary care surgery can be seen in Table 4.

Mean preoperative CDVA was 1.304 ± 0.794 logMAR and 1.289 ± 0.729 logMAR after surgery (*p* = 0.780). A vitreous hemorrhage was found in 24.5% and endophthalmitis in 1.4% of all included patients. Those two cases (1.4%) presented with post-traumatic endophthalmitis and one out of those two needed enucleation.

We subdivided all included patients into two categories. The first consisted of all patients with injuries involving the posterior segment (vitreous, retina, optic nerve, *n* = 90) and was compared to the second category of patients with injuries that did not affect structures of the posterior segment (*n* = 57). The postoperative CDVA in the first category was significantly lower than in the category of patients without involvement of the posterior segment (1.523 ± 0.654 logMAR vs. 0.944 ± 0.708 logMAR, *p* < 0.01). A further subdivision of patients with posterior segment involvement into those with only affected vitreous (*n* = 15) and those with affected retina and optic nerve (*n* = 75) revealed significantly better CDVA values for patients, whose posterior segment injuries had only affected the vitreous (1.133 ± 0.604 logMAR vs. 1.603 ± 0.638 logMAR, *p* = 0.013). Patients with open globe injuries with posterior segment involvement but only the vitreous had only tendentially but not significantly worse CDVA than patients without involvement of the posterior segment (1.133 ± 0.604 logMAR vs. 0.944 ± 0.708 logMAR, *p* = 0.308). Therefore, in our patients, posterior segment involvement including the retina or optic nerve is a negative predictive factor for the postoperative visual outcome.

## 4. Discussion

Open globe injuries are one of the most sight-threatening eye “diseases” in ophthalmology. Prior to the acute diagnosis and treatment of an open globe injury, a brief medical history should be performed to provide valuable information about the underlying responsible mechanisms and structural damages that have to be expected [17].

The spectrum of open globe injuries ranges from isolated corneal cuts to complex severe traumas involving various ophthalmic structures. Consequently, the prognosis for visual acuity also ranges from very good, such as in cases with only peripheral corneal defects, to a significantly reduced vision in cases with severe ocular trauma involving the posterior segments of the eye. Often, perforations of the cornea also affect the iris, lens and/or ciliary body. Permanent consequences can be traumatic mydriasis, iridodialysis or partial as well as total defects of the iris and secondary glaucoma [18,19]. The primary aim of the surgical treatment in those cases is a watertight re-adaptation of the wound edges with fine monofilament sutures (usually 10.0 nylon sutures). If the lens capsule is damaged in the process, the consequence is usually a traumatic cataract with the necessity for a lens exchange. Because the traumatic cataract is not always visible pre- or intraoperatively, the IOL replacement is performed in a second surgery after the acute phase. In severe blunt ocular trauma, common locations for ruptures of the globe are very often the area around the limbus, the thinnest parts of the sclera directly behind the insertion of the rectus muscles or preformed incisions from earlier ocular interventions. The primary aim of the surgical treatment in those cases is a rapid wound closure (e.g., with 7.0 vicryl sutures). Retinal detachment can occur directly due to the trauma or secondarily because of proliferation and subsequent traction of fibrovascular or vitreoproliferative tissue. The aim of surgical treatment in cases of retinal detachment is re-attachment of the retina, including ensured retinal holes or tears. This can be achieved with scleral buckling or with vitrectomy including the use of gas or, in some cases, silicone oil (Figure 3).

The appropriate antibiotic prophylaxis for cases of open globe injuries without presenting infection is controversial. In our clinic, we support the use of topical as well as systemic antibiotics (Cefuroxime 3 × 500 mg/d). Despite this precaution, we observed eight patients with post-traumatic endophthalmitis over the course of postoperative treatment and follow up observation [20].

The mean age of all included patients was 42.9 ± 22.2 years; the vast majority of the relatively young patients were male and at working age. Thus, long-term consequences such as lacking three-dimensional vision due to significantly reduced visual acuity with consequent restrictions regarding certain types of work and workplaces also have significant economic impacts beyond the health care system alone.

Open globe injuries are a very heterogeneous group of patients. This leads to mostly individual, unstandardized treatments and management of traumas. The prognosis of visual acuity depends on the mechanism of the trauma, the location of the wound and the damaged ocular structures.

In our analysis, patients with open globe injuries affecting the posterior segment had a significantly worse CDVA outcome than patients suffering from injuries not involving the posterior segment. This difference in CDVA was reduced to a non-significant trend if the posterior segment involvement was only the vitreous. Our interpretation of the evaluated data is that damaged retina or the optic nerve in open globe injuries are associated with a significantly worse postoperative CDVA outcome.

The prognosis after an open globe injury depends on numerous factors, such as the time to surgical care and the experience of the surgeon performing the procedure. The patients in this study were treated surgically as soon as possible and surgeries were not postponed to the normal working hours. As this is a retrospective study, the time from initial presentation to surgery was not recorded systematically and is not available for all cases.

One of the limitations is the retrospective character of our work, which allows for careful but still solid interpretations and conclusions. We present the data of a single center study in a university hospital and cases were collected over several years. Due to high security standards in working places and compulsory use of seatbelts, the incidence of open globe injuries could effectively be reduced in Germany. Another aspect is the heterogeneity of the trauma patients with individualized surgical approaches, making it difficult to classify and subdivide them into standardized subgroups. We tried to compensate for those limitations with a relatively large number of included patients over time and a simple classification into injuries with and without involvement of the posterior segment. Further analyses have to be carried out to evaluate the functional outcomes of those patients over longer time periods in order to better identify possible prognostic factors for a good visual outcome and/or following complications over time other than the initial involvement of the posterior segment.

In summary, we were able to identify retinal or optic nerve involvement as a negative prognostic factor for postoperative visual outcome in a large cohort of retrospectively analyzed patients with open globe injuries.

## Figures and Tables

**Table 1:** Calculating the Ocular Trauma Score (OTS).

Initial Visual Acuity in the Affected Eye	Raw Points	Variables		OTS Category	
>20/40	100 points	Globe rupture	Yes: 23 points/No: 0 points	Category 1	<45 points
20/50–20/200	90 points	Afferent pupillary defect (RAPD)	Yes: 10 points/No: 0 points	Category 2	45 to 65 points
19/200–1/100	80 points	Endophthalmitis	Yes: 17 points/No: 0 points	Category 3	66 to 80 points
Hand movement or light perception	70 points	Retinal detachment	Yes: 11 points/No: 0 points	Category 4	81 to 91 points
No light perception	60 points	Perforating globe injury	Yes: 14 points/No: 0 points	Category 5	92 to 100 points

For a predictive value regarding functional outcome, initial visual acuity and additional variables are calculated into the OTS. It is mainly used as standard for the assessment of ocular trauma in the Anglo-Saxon countries.

**Table 2:** Patients’ characteristics, age groups and percentage of work-related accidents.

No. of Subjects		*n* = 147
Gender	male	115 (78.2%)
	female	32 (21.8%)
Affected eye	Right	72 (49.0%)
	Left	75 (51.0%)
Age (years)	mean	42.9 ± 22.2 years
	1–20 (children and adolescents)	27 (18.4%)
	20–65 (working adults)	98 (66.7%)
	65 (seniors)	22 (15.0 %)
	Work-related accidents	54 (36.7%)
	non-work-related accidents	93 (63.3%)

**Table 3:** Classification of the open globe injury.

Type (Mechanism of injury)	A	Rupture	44 (29.9%)
	B	Penetration	58 (39.5%)
	C	Intraocular foreign body	38 (25.9%)
	D	Perforation	7 (4.8%)
Grade (initial visual acuity before surgery)	1	>20/40	29 (19.7%)
	2	20/50 to 20/100	23 (15.6%)
	3	19/100 to 5/200	25 (17.0%)
	4	4/200 to light perception	53 (36.1%)
	5	NLP	16 (10.9%)
	6	Not known	1 (0.7%)
Zone (location of the wound)		I Cornea (cornea +, sclera −)	76 (51.7%)
		II Limbus to 5 mm posterior into sclera (sclera +, NH −)	22 (15.0%)
		III posterior to 5 mm from limbus (sclera +, NH +)	47 (32.0%)
		Not known	2 (1.3%)
OTS Category		Category 1 (worse prognosis)	40 (27.2%)
		Category 2	46 (31.3%)
		Category 3	40 (27.2%)
		Category 4	21 (14.3%)
		Category 5 (best prognosis)	0

**Table 4:** Detailed injury characteristics and types of intraocular foreign body material.

Injured and damaged structures of the eye	Eyelids	26 (17.7%)
	Cornea	110 (74.8%)
	Iris	94 (63.9%)
	Lens	83 (56.5%)
	Sclera	71 (48.3%)
	Retina	70 (47.6%)
	Optic nerve	29 (19.7%)
	Vitreous	88 (59.9%) 59 (40.1%)
	Normal vitreousVitreous	36 (24.5%)
	HemorrhageEndophthalmitis	2 (1.4%)
	Vitreous loss	50 (34.0%)
Involvement of posterior segment	Yes	90 (61.2%)
	Vitreous only	15 (10.2%)
	Retina/Optic nerve	75 (51.0%)
	No	57 (38.8%)
Intraocular foreign body (IOFB) material	Metal	32 (84.2%)
	Glass	4 (10.5%)
	Plastic	0
	Organic material	2 (5.3%)
Foreign body removed successfully	Yes	36 (94.7%)
	No	2 (5.3%)
Surgical care of affected structures in the eye	Lid Yes/No	26/0
	Cornea Yes/No	107/3
	Sclera Yes/No	68/3
	Iris Yes/No	69/24, 1 not known
	Lens extraction Yes/No	62/21
	Vitrectomy Yes/No	71/17
	Retina Yes/No	47/23

Injuries with vs. without involvement of posterior segment.

## Data Availability

The data presented in this study are available on request from the corresponding author. The data are not publicly available due to data protection regulations.
